# Thyroid Storm Unmasked: A Rare Case of Acute COVID-19-Induced Thyroid Storm

**DOI:** 10.7759/cureus.45906

**Published:** 2023-09-25

**Authors:** Tate Pumphrey, Andrew Rizzo

**Affiliations:** 1 Emergency Medicine, Touro College of Osteopathic Medicine, Middletown, USA; 2 Emergency Medicine, New York City Health + Hospitals/South Brooklyn Health, Brooklyn, USA

**Keywords:** thyroid dysfunction, sars-cov-2, thyrotoxicosis, covid-19, thyroid storm

## Abstract

Thyroid storm is a severe form of thyrotoxicosis with a mortality rate of 8%-25% despite modern advancements in both early identification and treatment. It is more common in patients with a history of Graves’ disease; however, its incidence remains as low as 0.57-0.76 cases per 100,000 per year. In the setting of newly diagnosed COVID-19 with subsequent development of thyroid storm, the presentation is increasingly rare with only two reported cases in the current literature.

## Introduction

Since its emergence and progression to a pandemic, SARS-CoV-2 has been coupled with a plethora of disease processes. As of yet, infection with COVID-19 is linked to thyroid gland dysfunction, but there have been only two reported cases identified in the literature of COVID-19 triggering thyroid storm specifically [1]. The clinical distinction between COVID-19 symptoms and thyrotoxicosis can be obscure; therefore, heightened suspicion is required for timely diagnosis with the goal of preventing significant morbidity and mortality. We present a case of a critically ill patient with thyroid storm associated with COVID-19 infection.

## Case presentation

A 43-year-old female presented to the emergency department with a seven-day history of cough, shortness of breath, nasal congestion, myalgias, and chills starting shortly after traveling for work. The patient’s vital signs were as follows: heart rate of 132 beats per minute, blood pressure of 140/59 millimeters of mercury, respiratory rate of 24 breaths per minute, oxygen saturation of 96% on room air, and an oral temperature of 103.1°Fahrenheit. Her physical examination was notable for an uncomfortable, diaphoretic, and anxious-appearing female with an irregularly irregular heart rhythm and bilateral pedal edema.

The patient’s medical history included a diagnosis of Graves’ disease, for which she was currently receiving treatment with a prescribed maintenance dose of propylthiouracil at 100 mg three times daily. There had been no recent changes to her medication regimen, and no other relevant medical history or medication interactions were noted concerning her thyroid condition.

Initial resuscitation included a 2-liter bolus of lactated Ringer’s solution. In addition, 975 milligrams (mg) of oral acetaminophen, 200 mg of oral benzonatate, and 15 mg of intravenous (IV) ketorolac were administered. A full diagnostic laboratory workup was ordered, including complete blood count with differential, comprehensive metabolic panel, thyroid function tests, urinalysis, rapid SARS-CoV-2 nasal swab, N-terminal pro-B-type natriuretic peptide (NT-proBNP), and troponin level. Diagnostic imaging included a portable chest radiograph (CXR) and a 12-lead electrocardiogram (EKG).

The patient’s laboratory findings showed an undetectable thyroid-stimulating hormone and a free thyroxine of 5.65 nanograms per deciliter (ng/dL) (reference range: 0.80-1.70 ng/dL) along with a positive SARS-CoV-2 rapid nasal swab. Other results were significant for a proBNP level of 6,173 picograms per milliliter (pg/mL) (reference range: ≤299 pg/mL) and EKG demonstrating new-onset atrial fibrillation. The remainder of the patient’s laboratory results were unremarkable, and her CXR had no evidence of an acute pulmonary process. At this juncture, the patient’s Burch-Wartofsky score of 80 indicated thyroid storm (Table 1). In consultation with the hospitalist and intensivist, the patient would be admitted to the medical intensive care unit (MICU) and started on 80 mg of propranolol, 500 mg of propylthiouracil, 4 mg of IV dexamethasone, and 100 mg of IV doxycycline. She had a notable improvement in her symptoms and vital signs upon admission to the MICU. Cardiology was consulted following the incidental discovery of atrial fibrillation, but no interventions, such as cardioversion or rate control, were initiated. This decision was based on the understanding that once the thyroid disease was under control, the secondary effect of atrial fibrillation would resolve. Indeed, this turned out to be the case, and the patient was eventually transferred to a medical-surgical floor after spending 30 hours in the ICU. The patient was subsequently discharged home after a total of four days in the hospital with cardiology and endocrinology follow-up.

**Table 1:** Burch-Wartofsky Point Scale for the Diagnosis of Thyroid Storm Note: Original table Criteria and point scale adapted from Burch-Wartofsky Point Scale (BWPS) for thyrotoxicosis. (2023). Accessed: September 20, 2023: https://www.mdcalc.com/calc/3816/burch-wartofsky-point-scale-bwps-thyrotoxicosis#next-steps.

Criteria	Point Scale	Patient Score
Thermoregulatory dysfunction: temperature (°F)		Based on physical examination findings and vitals
99.0-99.9	5	
100.0-100.9	10	
101.0-101.9	15	
102.0-102.9	20	
103.0-103.9	25	+25
≥104.0	30	
Central nervous system dysfunction		
Absent	0	
Mild (agitation)	10	+10
Moderate (delirium, psychosis, extreme lethargy)	20	
Severe (seizures, coma)	30	
Gastrointestinal-hepatic dysfunction		
Absent	0	
Moderate (diarrhea, nausea/vomiting, abdominal pain)	10	+10
Severe (unexplained jaundice)	20	
Cardiovascular dysfunction		
Heart rate (beats/minute)		
<90	0	
90-109	5	
110-119	10	
120-129	15	
130-139	20	+20
≥140	25	
Congestive heart failure		
Absent	0	
Mild (pedal edema)	5	+5
Moderate (bibasilar rales)	10	
Severe (pulmonary edema)	15	
Atrial fibrillation		
Absent	0	
Present	10	+10
Consequence	Total score	Total score
Unlikely thyroid storm	<25	
Impending storm	25-44	
Thyroid storm	>45	80

## Discussion

COVID-19 (SARS-CoV-2) has been shown to predominately attach to the angiotensin-converting enzyme 2 (ACE2) receptor found on host cells via its spike protein [2]. Once attached, the virus can facilitate entry into host cells and exert damaging effects. Due to the thyroid’s dense population with the ACE2 receptor, it presents itself as a susceptible host to SARS-CoV-2 attack. Once inside the thyroid, the parafollicular and follicular epithelial cells are destroyed largely via apoptosis, causing the rupture of their contents into the bloodstream of patients [3]. This surge in thyroid hormone bound to protein or freely floating causes the downstream effects seen in thyrotoxicosis. According to studies in the current literature, it is not completely understood whether it’s the amount of thyroid hormone in the bloodstream or rather its acute elevation from normal to high levels that actually causes the thyroid storm [2]. Nevertheless, unchecked increased thyroid hormone has deleterious effects and increases morbidity and mortality in patients even without coexisting SARS-CoV-2 infection.

Although more and more research is coming out on the primary and secondary effects of SARS-CoV-2, it remains a novel virus. Thus, it is imperative for clinicians treating patients diagnosed with COVID-19 to operate with high suspicion of any life-threatening sequelae. As stated above, thyroid storm alone is associated with high mortality [4]. Coupled with SARS-CoV-2, the prognosis can worsen, considering evidence that TSH levels below the reference range of 0.34-4.80 mU/L, indicating overt thyrotoxicosis, increased mortality by up to 20% [5]. Therefore, patients with a history of thyroid disorder who appear toxic on presentation and have positive serology for SARS-CoV-2 should have thyroid function studies ordered routinely as they frequently develop thyrotoxicosis, and this drastically changes their hospital course and outcome [5,6].

Many patients may not associate thyroid disorders with the well-known warnings provided by the Centers for Disease Control and Prevention (CDC) over what chronic conditions put them at risk for a severe COVID-19 infection. Accordingly, patients diagnosed with Graves’ disease and those with a current or history of thyroid disorder should be informed that they are at added risk of not only severe COVID-19 infection but also potential complications such as thyrotoxicosis. Considering this and the fact that SARS-CoV-2 is trending toward a recurrent infectious virus, patients need to maintain adequate control of their disease. An underlying poorly controlled thyroid disorder might exacerbate a SARS-CoV-2 infection further [7].

It is clear how SARS-CoV-2 can act on the thyroid gland and some of the mechanisms proposed as to why this causes so much dysfunction. Future studies should focus on determining the correlation between levels of thyroid hormone and prognosis, allowing prompt treatment depending on risk stratification. One study was able to positively correlate the severity of SARS-CoV-2 infection and the decrease in TSH levels from normal [8]. This information could provide further insight to the treating physician on potential patient progression and therefore guide decision-making for prompt intervention and mortality aversion.

Although not discussed in this paper, the development of atrial fibrillation, as seen in this patient, was also detected in 32.3% of patients according to another study and offers yet another potential life-threatening sequelae of thyrotoxicosis caused by SARS-CoV-2 [5]. It can also be noted that along with SARS-CoV-2 preexisting blood clotting pathology, T4 has been shown to activate platelets. Elevated levels in thyrotoxicosis could exponentiate the pathological clotting that occurs in COVID-19 infection [9,10], further stressing the importance of adequate recognition and apt treatment of the underlying cause.

Table 1 outlines the Burch-Wartofsky Point Scale (BWPS), a numerical scoring system that assesses the severity of thyroid storm based on criteria related to various organ systems such as thermoregulatory, cardiovascular, gastrointestinal, and central nervous systems. Because thyroid storm carries a significant risk of mortality and morbidity, the BWPS employs lenient criteria to avoid missing potential cases, even if it occasionally results in false positives [11]. The BWPS serves as a valuable tool for clinicians to promptly and accurately identify and treat suspected cases of thyroid storm.

## Conclusions

Infection with SARS-CoV-2 has been linked to thyroid dysfunction, but this is just the third case of a patient displaying overt thyroid storm in the setting of acute COVID-19 infection. SARS-CoV-2 does not exclusively affect the thyroid and can have many other manifestations in other organs and tissues. Clinicians need to maintain high suspicion of the many life-threatening sequelae in thyroid disorder patients with recent COVID-19 diagnoses and should consider ordering routine thyroid studies to better assess and manage such critically ill patients.
